# Novel Implications in Molecular Diagnosis of Lynch Syndrome

**DOI:** 10.1155/2017/2595098

**Published:** 2017-01-29

**Authors:** Raffaella Liccardo, Marina De Rosa, Paola Izzo, Francesca Duraturo

**Affiliations:** ^1^Department of Molecular Medicine and Medical Biotechnology, University of Naples “Federico II”, 80131 Naples, Italy; ^2^CEINGE Biotecnologie Avanzate, University of Naples “Federico II”, 80131 Naples, Italy

## Abstract

About 10% of total colorectal cancers are associated with known Mendelian inheritance, as Familial Adenomatous Polyposis (FAP) and Lynch syndrome (LS). In these cancer types the clinical manifestations of disease are due to mutations in high-risk alleles, with a penetrance at least of 70%. The LS is associated with germline mutations in the DNA mismatch repair (MMR) genes. However, the mutation detection analysis of these genes does not always provide informative results for genetic counseling of LS patients. Very often, the molecular analysis reveals the presence of variants of unknown significance (VUSs) whose interpretation is not easy and requires the combination of different analytical strategies to get a proper assessment of their pathogenicity. In some cases, these VUSs may make a more substantial overall contribution to cancer risk than the well-assessed severe Mendelian variants. Moreover, it could also be possible that the simultaneous presence of these genetic variants in several MMR genes that behave as low risk alleles might contribute in a cooperative manner to increase the risk of hereditary cancer. In this paper, through a review of the recent literature, we have speculated a novel inheritance model in the Lynch syndrome; this could pave the way toward new diagnostic perspectives.

## 1. Introduction

Colorectal cancer (CRC) is a multifactorial disease in which genetic and environmental factors are involved. Familial CRC, in which one or more first-degree and/or second-degree relatives of the index case manifest CRC, constitutes approximately 20% of the total CRC burden [1]. High penetrance mutations confer a predisposition to CRC in the so-called hereditary syndromes, responsible for about 2–6% of the total CRC. Low penetrance mutations are found in the remaining part of CRC (about 96%), representing a risk factor in both sporadic and familial cases [2, 3]. CRC syndromes are defined on the basis of clinical, pathological, and, more recently, genetic findings [3] (Table 1).

Accordingly, the identification of predisposing genes allows for accurate risk assessment and more precise screening approaches. Lynch syndrome (LS) is the most common hereditary form of CRC with an incidence of 3–5% of all CRCs whereas its primary genetic counterpart, namely, Familial Adenomatous Polyposis (FAP), accounts for less than 1% of the total CRC burden [4].

LS and FAP are diseases with autosomal dominant inheritance, caused by germline mutations in the DNA mismatch repair genes (MMR) or in the Adenomatous Polyposis Coli tumor suppressor gene (APC), respectively. These syndromes may also occur in more attenuated forms. In FAP syndrome, attenuated forms (AFAP) are caused by low penetrance mutations (missense mutations) in the main APC gene or by biallelic loss of the MYH gene (MAP, MUTYH-associated polyposis with autosomal recessive inheritance), encoding a protein of the Base Excision Repair complex (BER) [5]. Variant clinic forms of LS are characterized by the presence of additional tumors of still unclear etiology in extracolonic locations. Recent studies suggest that an interaction between main genes (MMR) and modifier genes and/or environmental factors may beat the basis of these tumors. These variant syndromes include Muir-Torre syndrome (autosomal dominant) due to MSH2 and MLH1 genes mutations and characterized by the presence of cutaneous manifestations (multiple sebaceous adenomas, epithelioma, and keratoacanthoma) associated with colorectal and endometrial cancers and Turcot syndrome (autosomal dominant) associated with APC, PMS2, and MLH1 genes mutations and characterized by brain cancers (glioblastoma and cerebellar medulloblastoma) are associated with colorectal cancer [6, 7].

In the process of colorectal carcinogenesis many other genes are involved such as oncogenes and tumor suppressor genes that play a key role in the control of cell cycle. Mutations in these genes are at the basis of rarer inherited CRC syndromes. These are mainly “hamartomatous polyposis syndromes” characterized by the presence of benign adenomas arising from epithelial and/or stromal intestinal tissue, which increase the risk of developing CRC. These syndromes, whose characteristics are summarized in Table 1, include Peutz-Jeghers syndrome, juvenile polyposis, Cowden syndrome, and Bannayan-Riley-Ruvalcaba syndrome [8–11].

In this review we intend to highlight new insights into the molecular features of Lynch syndrome in favor of a novel inheritance model in contrast with the classical monogenic transmission; this could suggest new genetic and clinical surveillance approaches.

## 2. Lynch Syndrome (LS)

### 2.1. Genetics Features

LS is an autosomal dominant disease with recessive phenotype caused by a defect in one of the mismatch repair (MMR) genes. The main clinical-pathological features of the Lynch syndrome are as follows [1–11, 13]:

Autosomal dominant inheritance
Penetrance for colorectal cancer (CRC) of 85–90%
Earlier age of onset of CRC (~45 years ) with respect to general population (69 years)
Preferential tumor localization in the right-sided colon
Presence of multiple synchronous and metachronous colorectal cancers
Better prognosis than CRCs
Increased risk for extracolonic cancers
Accelerated carcinogenesis
Poorly differentiated tumors, with a marked lymphocytic peritumoral inflammation recalling features of the so-called “Crohn's reaction”
Microsatellite instability

The mismatch repair system was first studied in bacteria in which three proteins, MutS, MutL, and MutH, were identified. In humans, at least seven mismatch repair genes are involved in mismatch repair and their names derive from their structural homology to the bacterial proteins: the MutS homologues (MSH), MSH2 on chromosome 2p16, MSH3 on chromosome 5q11, and MSH6 on chromosome 2p16; the MutL homologues (MLH), MLH1 on chromosome 3p21 and MLH3 on chromosome 2p16; the postmeiotic segregation homologues (PMS), PMS1 and PMS2 on chromosome 7p22. No MutH homologues have been identified in humans [14]. MSH2 and MSH6 bind together to form a heteroduplex (MutSa) that predominantly identifies base pairs mismatched, while MSH2 and MSH3 (MutS*β*) combine to identify short insertions or deletions. MSH2 is essential for both complexes to function, while a functional overlap exists between MSH3 and MSH6. MLH1 and PMS2 (MutL*α*) or MLH3 (Mutl*γ*) also bind together to form a heteroduplex that interacts with MutS*α* or MutS*β* complex, stimulating excision and resynthesis of the abnormal DNA. Similarly to MSH2, also MLH1 is essential for both complexes to repair mismatches. Altogether, this group of four proteins recruits exonuclease-1 (EXO1), the proliferating cell nuclear antigen (PCNA), DNA polymerase (Pol*δ* or Pol*ε*), two replication factor (RPA and RFC), and a ligase, to repair DNA on the daughter strand at the mismatch point. If any of the four major proteins (MSH2, MLH1, MSH6, or PMS2) is functionally inactive, mismatches are not repaired [15, 16].

### 2.2. Microsatellite Instability

Consequently, a defective DNA MMR system increases the mutation rate and makes the cell vulnerable to mutations in genes controlling cell growth (tumor suppressor genes and oncogenes), resulting in an increased cancer risk.

In case of a defective MMR system, mutations occur frequently in small (usually mononucleotide or dinucleotide) repetitive DNA sequences, known as microsatellites. In MMR-deficient tumor cells the number of microsatellite repeat units can deviate from the corresponding normal DNA; the number of repeats is usually decreased even though it is occasionally found to be increased [17].

Length or size microsatellite variation is known as MSI (microsatellite instability). MSI (formerly referred to as MIN or, RER, replication error) is the molecular hallmark of LS since approximately 95% of all LS-associated cancers show MSI [13]. Although most microsatellite sequences are located in noncoding sequences (telomeres and centromeres), many genes contain repetitive sequences in their coding regions and some of these genes play key roles in the regulation of cell growth. Identification of an even growing number of guide genes and target genes of the mutator phenotype can lead to discover new complex molecular mechanisms that underlie the process of colorectal tumorigenesis [18].

MSI thereby serves as a reliable phenotypic marker of MMR deficiency in order to preselect patients eligible for germline mutation analysis in the MMR genes [19].

However, despite the fact that MSI is a reliable marker for MMR deficiency, however until 15% of sporadic CRCs showed an MSI phenotype. This is mainly caused by somatic hypermethylation of the MLH1-gene promoter. Methylation of the MSH2 promoter has also been reported but it is to be considered as a heritable somatic methylation because it is caused by a deletion of the last exon of EPCAM that is adjacent to MSH2 on chromosome 2 [18].

Hypermethylation of CpG islands in the MLH1 promoter (CIMP phenotype) causes severe inhibition of gene transcription thereby mimicking an inactivating gene mutation. If both copies of the gene are inactivated (biallelic hypermethylation), the MLH1 function is lost. This leads to microsatellite unstable cancers, especially in older patients. Therefore, in MLH1-deficient microsatellite unstable (MSI-H) tumors the MLH1 hypermethylation can be assessed to distinguish sporadic CRCs from LS-related cancers. Moreover, recent findings have also identified the BRAF gene as a marker to distinguish LS from sporadic cases of colon cancer [20, 21]. Indeed, an oncogenic BRAF mutation has been described only in one case among several LS tumors [22]. Specific activating mutations in the BRAF oncogene, usually the V600E missense mutation, can be detected in 40–87% of all sporadic microsatellite unstable tumors [13]. In this case, the survival in the subjects with MSI-H tumors and BRAF mutation is higher than sporadic tumors with BRAF mutation and MSI stable; therefore, the good prognosis of MSI-H tumors is not affected by the BRAF genotype [23]. Also in another study, the MSI-H/BRAF mutation group had a good prognosis [24]. This emphasizes even more the need to evaluate both BRAF mutation and MSI status in patients with CRC for an accurate prognosis [23]. These results indicate that in cases of MSI, BRAF mutations closely correlate with MLH1 promoter methylation in sporadic MSI CRCs, in contrast to LS characterized by germline mutations in the MMR genes.

In 1997 the National Cancer Institute recommended a panel, known as the “panel of Bethesda,” comprising five microsatellites: two mononucleotide repeats (BAT25, BAT26) and three dinucleotide repeats (D2S123, D17S250, D5S346) [25]. Tumors showing instability at two or more of these repeats (40% of markers) are defined at high instability (MSI-H); those with instability between 20–40% are classified as low instability (MSI-L) [26]; tumors without alteration (20% or less) are classified as stable (MSS). Subsequently, in order to improve the sensitivity rate and the predictive specificity, Bethesda guidelines were revised and other loci were enclosed in the panel test: BAT-25 and BAT-26 besides three other quasimonomorphic mononucleotide repeats, namely, NR21, NR22, and NR24 [27–29].

MSI testing is also very important because several pieces of evidence suggested that MSI-H tumors (stage II) are associated with a favorable prognosis when patients are not treated with 5-fluorouracil compared to MSI-L and MSS CRC [30, 31]. These different features are probably related to the lymphocytic infiltrate characteristic of MMR-deficient tumors that determines an antitumor immune response which may be abrogated by the immunosuppressive effects of the chemotherapy [17].

Besides MSI testing, analysis of MMR protein expression by immunohistochemistry (IHC) is routinely performed to identify patients with suspected Lynch syndrome. IHC testing is a specific (100%) and sensitive (92,3%) screening tool to identify MSI-H tumors [13, 32]. A more detailed discussion on MMR IHC is available in a recent review article [33].

### 2.3. Clinical Features

LS is characterized by a high lifetime risk for tumor development, especially in the case of CRC (20–70%), endometrial cancer (15–70%), and other extracolonic tumors (15%). These extracolonic malignancies include carcinomas of small intestine, stomach, pancreas and biliary tract, ovarium, brain, upper urinary tract, and skin. More recently, gastric cancers have been included in the tumor spectrum of LS [34]. The molecular and clinical-pathological profiles of gastric cancers in LS mutation carriers have been evaluated and compared with the profiles of sporadic gastric cancers, and several differences have been identified, while there are similarities with canonical HNPCC spectrum malignancies. Stomach can thus be considered as a target tissue in which somatic inactivation (“second hit”) of MMR genes may occur in carriers of a germline mutation (“first hit”) [34, 35].

### 2.4. Clinical Diagnosis of Lynch Syndrome

Identification of MMR gene mutation carriers is critical for improving cancer surveillance and effectiveness of prevention. Before MMR genes and their causal role in hereditary CRC cancer were identified, the International Collaborative Group on hereditary nonpolyposis colorectal cancer had established the Amsterdam criteria I in 1990. These criteria were used to identify families eligible for molecular analysis. Subsequently modified guidelines (Amsterdam criteria II) were designed to include extracolonic LS-related cancers [1]. Nevertheless, Amsterdam criteria resulted to be very restrictive and failed to identify a large portion of MMR gene mutation carriers. To overcome this issue, Bethesda guidelines, which were less restrictive and had a sensitivity greater than 90% even with a lower specificity (25%), were later defined [25].

## 3. New Insights into the Molecular Features of Lynch Syndrome

A long time, literature data report that in addition to the postreplicative repair, MMR proteins have developed various other functions that may have relevant roles in carcinogenesis [16]. These new roles include the following:

1. DNA damage signalling caused by exogenous carcinogens (heterocyclic amines, oxidative agents, and UV radiation) that is achieved through a synergistic action between the p53-homologous proteins (p53, p63 and p73) and the MutS*α*-MutL*α* complex; furthermore, in response to an exogenous damage, MLH1 interacts with the protein MRE11, a component of the “BRCA1 associated surveillance complex” (BASC), and regulates the cell cycle and the apoptotic pathway [36, 37]
2. Prevention of reparative recombination (gene conversion) between nonidentical sequences [38]
3. Promotion of meiotic crossover; several studies in* S. Cerevisiae *and knockout mice have shown that homologous chromosome recombination during meiosis is controlled by MMR proteins, in order to avoid mutational events due to deletions, insertions, or mismatched bases. Among the MMR proteins, MLH1, PMS2, and MLH3 are involved in this process. In fact, experimental murine deficiency of one of these three proteins is associated with male infertility (defective spermatogenesis) [38, 39]
4. Immunoglobulin diversification based on the “somatic hypermutation” (SHM) process, which is regulated by the MutS*α*-MutL*α* complex, in combination with two other proteins, AID (activation-induced cytidine deaminase) and Pol*μ* (DNA Polymerase “error-prone”) [40]; in particular, MutS*α* deficiency is associated with neoplastic transformation of T lymphocytes [41]
5. Expansion of repeated triplets (CTG, CGG) that underlie the pathogenesis of various neurodegenerative diseases such as Huntington's Disease, Myotonic Dystrophy, and Fragile X Syndrome. This mechanism is still unknown; however experimental evidence indicates that, although MutS*β* binds these expansions, the repair is prevented by looping conformations of these regions [42]. Since the triplet expansion is at the basis of the anticipation of the disease in the family, loss of function of MutS*β* may have a protective role against the intergenerational instability [43, 44]
6. Modulation of microRNA biogenesis by interaction of MMR proteins with the microprocessor complex; in particular, MutL*α* specifically binds pri-miRNAs and the complex Drosha/DGCR8 in order to stimulate the processing of pri-miRNAs to pre-miRNAs in a manner dependent on MutL*α* ATPase activity [45]

**Figure 1:**
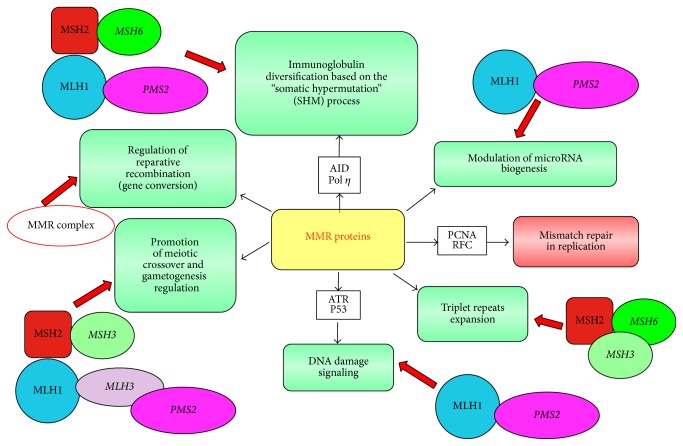
New role for MMR proteins.

These new features indicate that MMR deficiency strongly affects cellular resistance to reparative and/or apoptotic response to DNA damage because impairment of postreplicative MMR complexes associated with impairment of components of other cell systems (Figure 1).

## 4. Genotype-Phenotype Associations in Lynch Syndrome

### 4.1. Canonical Features

Germline mutations are distributed unevenly along each MMR gene, denoting the absence of mutational hot spots. Even the nature of the germline alterations is varied.

Absence of redundant functions for MSH2 and MLH1 proteins stresses the importance of these two genes; therefore, mutations in these genes are associated with aggressive forms of HNPCC, characterized by early age of onset, typically around 45 years of age, high penetrance, and high degree of microsatellite instability (MSI-H) [46]. The CRC incidence is similar in subjects with mutations in MLH1 and MSH2 (84% and 71% resp.); however, individuals with alterations in the MSH2 gene show a higher incidence (48–61%) of extracolonic malignancy (endometrial, gastric, ovarian, and kidney cancer) than those carrying mutations in the MLH1 gene (11–42%) [47].

The clinical phenotype is different when minor genes are involved. Mutations in MSH6, for example, seem to cause a form of “attenuated” HNPCC, characterized by lower penetrance, later age of onset, usually around 60 years of age, and MSI-L [48].

Defects in the PMS2 gene are instead associated with early tumor development and microsatellite instability, although some features are different with respect to cancers caused by the MLH1 and MSH2 mutations. PMS2 mutations are associated with combined presence of multiple colorectal adenomas and glioblastomas (Turcot syndrome). The specificity of brain tumor is probably linked to the accumulation of mutations in target genes (oncogenes, tumor suppressor) more specifically expressed in the brain [49]. Recently, MLH3 variants were associated with brain cancer predisposition [50].

In the MSH3 gene, missense, silent, and intronic variations have been mainly identified; these mutations are associated with a severe phenotype in the case they are inherited in combination with each other or associated with variants in the MSH2 gene [51]. In fact, MSH3 knockout mice showed a low susceptibility to cancer development that caused late-onset colorectal cancer, whereas double mutant MSH3-MSH6 mice showed a very similar phenotype to that found in mice lacking MSH2. These results are justified by the redundant function of the MSH3 and MSH6 genes [52]. Moreover, MSH3 inactivation is primarily associated with instability of tetranucleotide repeats (EMAST) that has been frequently observed in moderately or poorly differentiated adenocarcinomas as well as in other cancers including lung, kidney, ovarian, and bladder cancer [14, 53].

In recent years, numerous studies have found an association between the development of hematopoietic and intestinal tumors in infant age and the presence of homozygous mutations in the MLH1, MSH2, MSH6, and PMS2 genes [54, 55]. This phenotype was also associated with heterozygous mutations in two or more MMR genes, suggesting a mechanism of compound heterozygosity [56–58].

In a subset of LS patients, a germline mutation at the 3′ end of the EPCAM (TACSTD1) gene has been identified resulting in allelic-specific methylation and transcription silencing of MSH2, which is located upstream of the EPCAM gene. The EPCAM gene encodes for the Epithelial Cell Adhesion Molecule protein that is involved in cell signalling, migration, proliferation, and differentiation. Accordingly, this mutation may contribute to the development of extracolonic cancers [59].

### 4.2. Noncanonical Features

Recently a group of Lynch-like syndrome patients was described [60]. This group may account for as much as 70% of suspected Lynch syndrome subjects. Unlike sporadic MSI cancer, Lynch-like patients are nearly impossible to differentiate from Lynch patients; they are MSI-positive and cancer tissues express abnormal MMR protein, not only for MLH1 as in sporadic MSI cancers but also for the other MMR proteins, such as MSH2, MSH6, and PMS2, as in Lynch syndrome cancers. Lynch-like patients show a mean age of onset comparable to LS. The only differentiating features between these two syndromes are the lower incidence for CRC and other LS-associated cancers and the absence of MMR genes germline mutation in Lynch-like syndrome. There are likely three potential reasons for cancer onset in Lynch-like patients: (a) a genetic process within the tumors other than germline mutations coupled with second allele inactivation, (b) unknown germline mutations in other genes than the DNA MMR genes that can drive MSI, and/or (c) unidentified germline mutations in the DNA MMR genes [12, 61].

Mensenkamp et al. [62] noted that a considerable number of MSI-positive tumors lack any known molecular mechanism for their development. Patients were screened for somatic mutations and for loss of heterozygosity in MLH1 and MSH2 genes. This research identified two somatic mutations in 13 of 25 tumors, 8 of which were MLH1-deficient and 5 were MSH2-deficient, indicating that such acquired mutations underlie more than 50% of the MMR-deficient tumors that have not been found associated with germline mutations or promoter methylation. This is in contrast with LS that is associated with germline mutations in the MMR genes.

Moreover, other hereditary factors might play a role in tumor development. For example, deletions affecting genes that regulate MSH2 degradation were shown to lead to MMR deficiency and undetectable levels of MSH2 protein. Moreover, cells lacking SETD2 (H3K36 trimethyltransferase SET domain containing protein 2) display MSI due to the loss of an epigenetic histone mark that is essential for the recruitment of the MSH2-MSH6 complex. Whether these mechanisms lead to MSH2-deficient colorectal cancer remains to be clarified [63].

In these cases high-throughput sequencing procedures play an important role to identify new constitutive and somatic mutations in putative genes associated with hereditary predisposition to cancer [64].

It is also noteworthy that, in addition to canonical inactivation via gene mutation, MMR activity can also be modulated by changes in MMR gene expression. This type of alteration may be the result of mutations occurring in regions that are not always routinely analyzed such as the promoter and the 5′ and 3′-untraslated regions.

Previous studies have defined and characterized the core promoter regions of hMSH2 (from −300 to −17 upstream of the start codon) [65] and hMLH1 (from −220 to −39 upstream of the start codon) [66]; subsequent studies have been carried out to demonstrate that germline mutations in these regions are involved in LS [67, 68].

Regarding mutations in the 3′UTR of MMR genes, a 3-nucleotide (TTC) deletion in the MLH1 3′UTR was found in leukemia patients [69]. This alteration was shown to destroy a binding site for miR-422a and there is a downregulation suggesting a possible role for the miRNA in regulation of MLH1expression [69].

Therefore, cell levels of MMR are likely to be under a tight regulation in order to prevent the overproduced protein which may sequester other factors involved in controlling the mutation rate. Potentially adverse consequences of overproduced MLH1 and MSH2 are highlighted by a report showing that apoptosis is induced in a human cell line when these two genes were expressed under the control of the cytomegalovirus (CMV) promoter [70]. The dangerous excess of MMR protein can also be the effect of homodimerization complex as shown by a study in yeast cells of Shcherbakova et al. [71] showing that the MLH1-MLH1 homodimer replaced the MLH1-PMS1/PMS2/MLH3 heterodimer, inactivating also the MutS*α* and MutS*β* functions, thus resulting in nonfunctional MMR complex.

This concept is also partially extended to other minor MMR genes; overexpression of the MSH3 gene in cultured mammalian cells selectively inactivates MutS*α* because MSH2 is sequestered into a MSH2-MSH3 (MutS*β*) complex, resulting in reduced MutS*α*-dependent repair of base-base mismatches and a strong base substitution mutator phenotype [72].

Finally, several MSI tumors with unknown cause of MMR inactivation could display a miRNA down- or upexpression genotype that specifically modulate MMR genes [73, 74]. miRNA expression is in turn regulated by DNA damage [75]. miRNAs able to regulate the mismatch repair function are miR-155 and miR-21 that significantly downregulate the core MMR proteins, MSH2, MSH6, and MLH1, and have been associated with a mutator phenotype, in particular with MSI inflammatory bowel diseases (IBD) CRCs [76, 77].

## 5. Characterization of the “Variants of Uncertain Significance” (VUS) in the MMR Genes

Several mutations identified in the MMR genes are missense, silent, or intronic variants. The influence of these variants on the development of cancer is often a controversial topic; therefore they are classified as “VUS,” Variant of Uncertain Significance [78, 79].

Several criteria can be applied to assess the possible pathogenicity of a VUS, [57, 80]; these criteria are as follows: (1) de novo appearance; (2) segregation with the disease; (3) absence in normal individuals; (4) change of amino acid polarity or size; (5) occurrence of the amino acid change in a domain that is evolutionary conserved between species and/or shared between proteins belonging to the same protein family (in silico analysis); (6) effects on splicing or on protein function; (7) loss of the nonmutated allele due to a large deletion in the tumor DNA (loss of heterozygosity (LOH)); (8) loss of protein expression in the tumor; (9) evaluation of MSI in tumor tissues. All studies conducted to date show that none of the above criteria, including functional assays, is an indicator of pathogenicity, if taken alone; it is necessary that a combination of strategies be used in order to lead to a correct assessment of the pathogenicity of uncertain variants [81]. According to these observations, a classification of MMR sequence variants identified by genetic testing has been proposed based on a 5-class system, using a multifactorial likelihood model (Table 2).

Variant-Class 5 includes coding sequence variation resulting in a stop codon (nonsense or frameshift), splicing aberration variants by mRNA assay, large genomic deletions or duplications, abrogated mRNA/protein function variants based either on laboratory assays, on evidence for cosegregation with disease, and on MSI tumor, and/or loss of MMR protein expression.

Variant-Class 4 includes IVS+-1 or IVS+-2 mutations resulting in splicing aberrations, variants abrogating mRNA/protein function based on laboratory assays, evidence of cosegregation with disease or MSI tumor, and/or loss of MMR protein expression.

Variant-Class 3 includes large genomic duplications, missense alterations, small in-frame insertions/deletions, silent variants, intronic variants, and promoter and regulatory region variants for which insufficient molecular evidence is available and with intermediate clinical effects or low penetrance alleles.

Variant-Class 2 includes synonymous substitutions and intronic variants with no associated mRNA aberration, with a proficient protein expression/function, and lack of cosegregation and/or MSS tumor.

Variant-Class 1 includes variants reported in control reference groups and excluded as founder pathogenic sequence variant.

According to this classification, most of the VUS tested for the MMR genes are likely to be pathogenetic and thus they can be associated with the HNPCC phenotype.

For the MLH1 gene, 52 out of 73 VUS resulted to be pathogenetic (70%), similar pathogenicity has been demonstrated for 25 out of 35 VUS identified in the MSH2 gene, (71%) (https://www.insight-group.org).

For minor MMR genes, the percentage of pathogenic VUSs is reduced due to the milder mutational contribution of these genes to the development of the disease. For the MSH6 gene, only 1 out of 8 variants studied (13%) was found to have aberrant effects on protein function; for the PMS2 gene, 4 variants were analyzed and all (100%) seem to have a causative role in Lynch syndrome; for the MLH3 gene, however, functional assays have not identified any variant with certain pathogenetic significance; finally, for the MSH3 gene relevant functional studies have not yet been reported (https://www.insight-group.org).

## 6. Probability of a “Synergistic Effect” between Low Risk Allelic Variants in the MMR Genes

With the advent of high-throughput technologies it is becoming even more possible to analyze a great number of polymorphic variants in large cohorts of cases and controls of specific cancers, such as breast, prostate, and colorectal cancer, thus providing new insights into common mechanisms of carcinogenesis. In some cases, VUSs make a more substantial overall contribution to cancer risk than the well-assessed severe Mendelian variants. It is also possible that the simultaneous presence of some polymorphisms and VUSs in cancer predisposition genes that behave as low risk alleles might contribute in a cooperative manner to increase the risk of hereditary cancer [12, 64]. Therefore, current literature data suggest a significant proportion of the inherited susceptibility to relatively common human diseases may be due to the addition of the effects of a series of low frequency variants of different genes, probably acting in a dominant and independent manner, with each of them conferring a moderate but even detectable increase in the relative cancer risk [50, 51, 75, 81–83]. Therefore, several functional studies based on GWAS data related to cancer susceptibility have been performed in an attempt to demonstrate the effective association and to test the hypothesis of synergistic effects between low risk allelic variants [80, 81].

In a recent study on yeast genome, it has been shown that the minor alleles of the MMR complex cause a weak mutator phenotype; however, their interaction causes a more severe mutator phenotype [82]. In this study, 11 polymorphisms and 14 missense variants of uncertain significance previously identified in the MSH2, MLH1, MSH6, and PMS2 genes were studied by complementation tests. The mutator effect of these variants was tested singly and in combination with each other.

In 2011, Kumar et al. showed that some variants occurring in domain I of the MSH2 gene in yeast strains (msh2Δ1) behave as weak alleles in the presence of a functional protein MSH6, as they do not alter the stability of the MutS*α* complex. However, by combining these variants with weak alleles falling in the N-terminal region (NTR) (DNA binding domain) of the MSH6 gene, a strong mutator phenotype was found. Moreover, the mutator synergistic effect is also found between different systems of DNA damage response. A recent population study by Smith et al. [83] has shown that the simultaneous presence of mutations in the TP53 gene and single nucleotide polymorphisms (SNPs) in genes belonging to different repair systems as BER, NER, MMR, and DSBR (Double-Strand Break Repair) complex is associated with an earlier age of onset of breast cancer (<50 years). Therefore, in this case, the authors suggest an additive or multiplicative effect.

The additive effect of low penetrance genes could also be the cause of atypical Lynch syndromes such as familial CRC type X [84]. With respect to LS, the familial CRC type X is more often located in the distal colon; extracolonic cancers are less frequent than in LS, and the age of onset is delayed. The sine qua non condition for this diagnosis is the absence of molecular genetic evidence of LS (MSI, IHC, or MMR mutations).

## 7. Scientific Hypothesis and Our Results

Molecular characterization of patients with a clinical diagnosis of Lynch syndrome currently relies on the identification of point mutations and large rearrangements [85, 86] by DHPLC and MLPA, respectively, in the major MMR genes, MLH1 and MSH2.

This strategy does not always provide informative results for genetic counseling. Indeed, many families selected according to international diagnostic criteria (Amsterdam Criteria and Bethesda Guidelines) do not have a molecular diagnosis of Lynch syndrome. In our experience, we have identified several patients carrying genetic VUS (missense, intronic, and silent variants) not only in main genes, MLH1 and MSH2 [80], but also in other MMR genes. According to international recommendations (Colon cancer Family Registry 2009, InSiGHT Variant Interpretation Committee 2011) we used a multifactorial likelihood model in an attempt to define a pathogenetic role for numerous VUS identified in MMR genes [50, 51, 64, 85]. The segregation analysis, population studies (to exclude the polymorphic nature of the variant), assessment of MSI in tumor tissues, detection of loss of protein expression in tumor tissues by immunohistochemical analysis (IHC), in silico analysis by a variety of bioinformatics tools, and gene expression studies are strategies that have to be used to assess an exhaustive evaluation of the pathogenicity of uncertain variants [85].

In light of literature data indicated that “minor” MMR proteins have other functions besides the postreplicative repair that could be highly relevant in carcinogenesis; in our laboratories, we have also analyzed the minor MMR genes, MSH6, PMS2, MLH3, and MSH3 for germline variants detected in patients negative for germline mutations in the major MMR genes. Many of the subjects analyzed in our series [50, 51, 64, 85] showed coinheritance of different genetic alterations in the minor MMR genes (Table 3) we speculate a likely additive role of low penetrance alleles in the disease development, in favor of a putative polygenic inheritance for Lynch syndrome, according to recent literature data [87–89].

## 8. Conclusion

The recent literature data describe the MMR proteins increasingly new roles. It is now known that MMR proteins not only have an exclusive role in the repair of DNA mismatch but are also involved in many other processes relevant in carcinogenesis. Therefore, some genetic variants may not affect the repair function but may be responsible for the loss of other important functions related to MMR proteins. In the light of these new roles of the MMR proteins it is essential to widen the mutations detection in all genes that are part of MMR complex. This will lead to the identification of numerous VUS in these genes. However, the study of VUS identified in MMR genes provides important information on the pathogenicity of the many genetic variants that are identifying in patients with suspected diagnosis of Lynch syndrome. Very often these variants are causing the disease, perhaps with a different degree of pathogenicity. Sometimes the simultaneous presence of molecular alterations in several MMR genes could be causing the onset of tumor. All these reassess the classical model of monogenic transmission in favor of a polygenic inheritance of Lynch syndrome.

Therefore, these recent findings allow clarifying better the genotype-phenotype correlations in Lynch syndrome, demonstrating the importance of molecular analysis to improve the genetic counseling and, consequently, the clinical surveillance.

## Figures and Tables

**Table 1:** Hereditary colon cancer syndromes: clinical and genetic features. (AD, autosomal dominant; AR, autosomal recessive).

Disease (OMIM)	Gene	Incidence	Inheritance	Mutation identified (%)	Penetrance	Clinical features
*Hereditary nonpolyposis colorectal cancer (HNPCC) (114500)*	MLH1, MSH2, MSH6, PMS2, MLH3, EPCAM	1 in 400	AD	Point mutation, large rearrangements (60–80%)	90%	Proximal CRC, endometrial carcinoma, ovarian tumors, small bowel carcinoma, urinary tract carcinoma

*Classical familial adenomatous polyposis (FAP)(175100)*	APC	1 in 8000	AD	Point mutation, large rearrangements (80–90%)	<100%	100 to >500 adenomatous polyps of large bowel, duodenum, stomach

*Attenuated FAP (AFAP) (175100)*	APC	<1 in 8000	AD	Point mutation, large rearrangements (20–30%)	<100%	10 to 100 adenomatous polyps of large bowel, duodenum, stomach

*MUTYH-associated polyposis (MAP) (608456)*	MUTYH	<1 in 10000	AR	Point mutation, large rearrangements (15–20%)	<100%	20 to 100 adenomatous polyps of large bowel, duodenum, stomach

*Muir Torre syndrome (HNPCC) (158320)*	MLH1, MSH2	<1 in 400	AD	Point mutation, large rearrangements (60–80%)	90%	CRC, endometrial carcinoma multiple sebaceous adenomas, epithelioma, keratoacanthoma

*Turcot syndrome (HNPCC) (276300)*	APC, PMS2, MLH1	<1 in 400	AD	Point mutation, large rearrangements (60–80%)	90%	CRC, glioblastoma, cerebellar medulloblastoma

*Peutz-Jeghers syndrome (PJS) (175200)*	STK11 (LKB1)	1 in 200000	AD	Point mutation, large rearrangements (90%)	95–100%	<20 juvenile polyps (PJ) of large bowel, duodenum, stomach, mucocutaneous/perioral hyperpigmentation, ovarian tumors, breast cancer

*Juvenile polyposis syndrome (JPS) (174900)*	SMAD4, BMPR1A	1 in 100000	AD	Point mutation, large rearrangements (60%)	90–100%	5 to 100 JP of large bowel, duodenum, stomach, gastric cancer

*Cowden syndrome (CS) (158350)*	PTEN	1 in 200000	AD	Point mutation, large rearrangements (80%)	90–95%	Multiple JP/lipomas of large bowel, duodenum, stomach, mucocutaneous tumors, breast cancer, endometrial carcinoma, thyroid cancer

*Bannayan-Ruvalcaba-Riley syndrome (BRRS) (153480)*	PTEN	1 in 200000	AD	Point mutation, large rearrangements (60%)	90–95%	Multiple JP/lipomas of large bowel, duodenum, stomach, microcephaly, developmental delay, hemangiomatosis

**Table 2:** Proposed classification system for MMR variant interpretation *(Colon Cancer Family Registry 2009, InSiGHT Variant Interpretation Committee 2011)*.

Class	Description	Probability of being pathogenic
5	Definitely pathogenic	>0.99
4	Likely pathogenic	0.95–0.99
3	Uncertain	0.05–0.949
2	Likely not pathogenic or of little clinical significance	0.001–0.049
1	Not pathogenic or of no clinical significance	<0.001

**Table 3:** Patients carrying variants in several MMR genes: MSH6, PMS2, MSH3, and MLH3; ^*∗*^patient 504 showing also the UV in MSH2 gene (c.984 C>T) [64].

*Patients*	*MSH6*	*PMS2*	*MLH3*	*MSH3*	*Phenotype*
9525	ex4 c.2633 T>C (Val>Ala)	ex14 c.2324 A>G (Asn>Ser)	ex1 c.2530 C>T (Pro>Ser) c.2533 T>C (Ser>Pro)	IVS7 -9 T>C	Amsterdam +

013		ex6 c.665G>C (Ser>Thr) IVS6 +16A>G	ex1 c.2533 T>C (Ser>Pro)		No Amsterdam MSI-H

103	ex5 c.3261_62insC (Phe>stop)		ex1 c.2533 T>C (Ser>Pro)	ex12 c.1860G>A (Asp>Asn)	No Amsterdam later onset MSI-H

423		IVS12-4G>A	ex1 c.2530 C>T (Pro>Ser) c.2533 T>C (Ser>Pro)		Amsterdam + later onset MSI-L

015	ex5 c.3295_97delTT (Ile>stop)		ex1 c.666 G>A (Lys) c.2191 G>T (Val>Phe) c.2533A>G (Ser>Gly)		Amsterdam + MSI-H

210	ex4 c.2941 A>G (Ile>Val)	IVS6+16A>G ex13 c.2324 T>C (Phe)	ex1 c.2530 C>T (Pro>Ser)	IVS6-64 C>T	Amsterdam + MSI not detected

211	ex4 c.2941 A>G (Ile>Val)	IVS12-4 G>A		IVS6-64 C>T	Amsterdam + MSI not detected

416		ex11 c.1714C>A (Thr>Lys)	ex 1 c.2027G>A (Arg>Lys)	IVS6-64 C>T	Amsterdam + MSI-H

504^*∗*^				ex4 c.693G>A (Pro) ex20 c.2732 T>G (Leu>Trp)	Amsterdam + MSI-H

## References

[1] Lynch H. T., Drescher K., Knezetic J., Lanspa S. (2014). Genetics, biomarkers, hereditary cancer syndrome diagnosis, heterogeneity and treatment: a review. *Current Treatment Options in Oncology*.

[2] Chung C. C., Chanock S. J. (2011). Current status of genome-wide association studies in cancer. *Human Genetics*.

[3] Valle L. (2014). Genetic predisposition to colorectal cancer: where we stand and future perspectives. *World Journal of Gastroenterology*.

[4] De Rosa M., Dourisboure R. J., Morelli G. (2004). First genotype characterization of Argentinean FAP patients: identification of 14 novel APC mutations. *Human Mutation*.

[5] De Rosa M., Galatola M., Borriello S., Duraturo F., Masone S., Izzo P. (2009). Implication of adenomatous polyposis coli and MUTYH mutations in familial colorectal polyposis. *Diseases of the Colon and Rectum*.

[6] Samadder N. J., Jasperson K., Burt R. W. (2015). Hereditary and common familial colorectal cancer: evidence for colorectal screening. *Digestive Diseases and Sciences*.

[7] De Rosa M., Pace U., Rega D. (2015). Genetics, diagnosis and management of colorectal cancer (Review). *Oncology Reports*.

[8] Carlomagno N., Duraturo F., Candida M. (2015). Multiple splenic hamartomas and familial adenomatous polyposis: a case report and review of the literature. *Journal of Medical Case Reports*.

[9] Galatola M., Paparo L., Duraturo F. (2012). Beta catenin and cytokine pathway dysregulation in patients with manifestations of the “PTEN hamartoma tumor syndrome”. *BMC Medical Genetics*.

[10] Paparo L., Rossi G. B., Delrio P. (2013). Differential expression of PTEN gene correlates with phenotypic heterogeneity in three cases of patients showing clinical manifestations of PTEN hamartoma tumour syndrome. *Hereditary Cancer in Clinical Practice*.

[11] Galatola M., Miele E., Strisciuglio C. (2013). Synergistic effect of interleukin-10-receptor variants in a case of early-onset ulcerative colitis. *World Journal of Gastroenterology*.

[12] Carethers J. M. (2014). Differentiating lynch-like from lynch syndrome. *Gastroenterology*.

[13] van Lier M. G. F., Wagner A., van Leerdam M. E. (2010). A review on the molecular diagnostics of Lynch syndrome: a central role for the pathology laboratory. *Journal of Cellular and Molecular Medicine*.

[14] Hegan D. C., Narayanan L., Jirik F. R., Edelmann W., Liskay R. M., Glazer P. M. (2006). Differing patterns of genetic instability in mice deficient in the mismatch repair genes Pms2, Mlh1, Msh2, Msh3 and Msh6. *Carcinogenesis*.

[15] Jun S.-H., Kim T. G., Ban C. (2006). DNA mismatch repair system: classical and fresh roles. *FEBS Journal*.

[16] Jiricny J. (2006). The multifaceted mismatch-repair system. *Nature Reviews Molecular Cell Biology*.

[17] Sinicrope F. A., Sargent D. J. (2012). Molecular pathways: microsatellite instability in colorectal cancer: prognostic, predictive, and therapeutic implications. *Clinical Cancer Research*.

[18] Alhopuro P., Sammalkorpi H., Niittymäki I. (2012). Candidate driver genes in microsatellite-unstable colorectal cancer. *International Journal of Cancer*.

[19] Zaanan A., Meunier K., Sangar F., Fléjou J.-F., Praz F. (2011). Microsatellite instability in colorectal cancer: from molecular oncogenic mechanisms to clinical implications. *Cellular Oncology*.

[20] Imai K., Yamamoto H. (2008). Carcinogenesis and microsatellite instability: the interrelationship between genetics and epigenetics. *Carcinogenesis*.

[21] Sharma S. G., Gulley M. L. (2010). BRAF mutation testing in colorectal cancer. *Archives of Pathology and Laboratory Medicine*.

[22] Wang L., Cunningham J. M., Winters J. L. (2003). BRAF mutations in colon cancer are not likely attributable to defective DNA mismatch repair. *Cancer Research*.

[23] Nakaji Y., Oki E., Nakanishi R. (2017). Prognostic value of BRAF V600E mutation and microsatellite instability in Japanese patients with sporadic colorectal cancer. *Journal of Cancer Research and Clinical Oncology*.

[24] Lochhead P., Kuchiba A., Imamura Y. (2013). Microsatellite instability and BRAF mutation testing in colorectal cancer prognostication. *Journal of the National Cancer Institute*.

[25] Boland C. R., Thibodeau S. N., Hamilton S. R. (1998). A National Cancer Institute workshop on microsatellite instability for cancer detection and familial predisposition: development of international criteria for the determination of microsatellite instability in colorectal cancer. *Cancer Research*.

[26] Vilar E., Mork M. E., Cuddy A. (2014). Role of microsatellite instability-low as a diagnostic biomarker of Lynch syndrome in colorectal cancer. *Cancer Genetics*.

[27] Xicola R. M., Llor X., Pons E. (2007). Performance of different microsatellite marker panels for detection of mismatch repair-deficient colorectal tumors. *Journal of the National Cancer Institute*.

[28] Umar A., Boland C. R., Terdiman J. P. (2004). Revised Bethesda Guidelines for hereditary nonpolyposis colorectal cancer (Lynch syndrome) and microsatellite instability. *Journal of the National Cancer Institute*.

[29] Suraweera N., Duval A., Reperant M. (2002). Evaluation of tumor microsatellite instability using five quasimonomorphic mononucleotide repeats and pentaplex PCR. *Gastroenterology*.

[30] Sargent D. J., Marsoni S., Monges G. (2010). Defective mismatch repair as a predictive marker for lack of efficacy of fluorouracil-based adjuvant therapy in colon cancer. *Journal of Clinical Oncology*.

[31] Kim J. H., Kang G. H. (2014). Molecular and prognostic heterogeneity of microsatellite-unstable colorectal cancer. *World Journal of Gastroenterology*.

[32] Lynch H. T., Lynch P. M., Lanspa S. J., Snyder C. L., Lynch J. F., Boland C. R. (2009). Review of the Lynch syndrome: history, molecular genetics, screening, differential diagnosis, and medicolegal ramifications. *Clinical Genetics*.

[33] Shia J., Holck S., Depetris G., Greenson J. K., Klimstra D. S. (2013). Lynch syndrome-associated neoplasms: a discussion on histopathology and immunohistochemistry. *Familial Cancer*.

[34] Corso G., Marrelli D., Roviello F. (2011). Familial gastric cancer: update for practice management. *Familial Cancer*.

[35] Gylling A., Abdel-Rahman W. M., Juhola M. (2007). Is gastric cancer part of the tumour spectrum of hereditary non-polyposis colorectal cancer? A Molecular Genetic Study. *Gut*.

[36] O'Brien V., Brown R. (2006). Signalling cell cycle arrest and cell death through the MMR System. *Carcinogenesis*.

[37] Yamane K., Schupp J. E., Kinsella T. J. (2007). BRCA1 activates a G2-M cell cycle checkpoint following 6-thioguanine-induced DNA mismatch damage. *Cancer Research*.

[38] Nicholson A., Hendrix M., Jinks-Robertson S., Crouse G. F. (2000). Regulation of mitotic homeologous recombination in yeast: functions of mismatch repair and nucleotide excision repair genes. *Genetics*.

[39] Ji G., Long Y., Zhou Y., Huang C., Gu A., Wang X. (2012). Common variants in mismatch repair genes associated with increased risk of sperm DNA damage and male infertility. *BMC Medicine*.

[40] Roa S., Li Z., Peled J. U., Zhao C., Edelmann W., Scharff M. D. (2010). MSH2/MSH6 complex promotes error-free repair of AID-Induced dU:G mispairs as well as error-prone hypermutation of A:T Sites. *PLoS ONE*.

[41] Jiang C., Zhao M.-L., Waters K. M., Diaz M. (2012). Activation-induced deaminase contributes to the antibody-independent role of B cells in the development of autoimmunity. *Autoimmunity*.

[42] Tomé S., Holt I., Edelmann W. (2009). ATPase domain mutation affects CTG^∗^CAG repeat instability in transgenic mice. *PLoS Genetics*.

[43] Dragileva E., Hendricks A., Teed A. (2009). Intergenerational and striatal CAG repeat instability in Huntington's disease knock-in mice involve different DNA repair genes. *Neurobiology of Disease*.

[44] Seriola A., Spits C., Simard J. P. (2011). Huntington's and myotonic dystrophy hESCs: down-regulated trinucleotide repeat instability and mismatch repair machinery expression upon differentiation. *Human Molecular Genetics*.

[45] Mao G., Pan X., Gu L. (2008). Evidence that a mutation in the MLH1 3′-untranslated region confers a mutator phenotype and mismatch repair deficiency in patients with relapsed leukemia. *Journal of Biological Chemistry*.

[46] Hsieh P., Yamane K. (2008). DNA mismatch repair: molecular mechanism, cancer, and ageing. *Mechanisms of Ageing and Development*.

[47] Koornstra J. J., Mourits M. J., Sijmons R. H., Leliveld A. M., Hollema H., Kleibeuker J. H. (2009). Management of extracolonic tumours in patients with Lynch syndrome. *The Lancet Oncology*.

[48] Lucci-Cordisco E., Rovella V., Carrara S. (2001). Mutations of the 'minor' mismatch repair gene MSH6 in typical and atypical hereditary nonpolyposis colorectal cancer. *Familial Cancer*.

[49] Chao E. C., Lipkin S. M. (2006). Molecular models for the tissue specificity of DNA mismatch repair-deficient carcinogenesis. *Nucleic Acids Research*.

[50] Duraturo F., Liccardo R., Izzo P. (2016). Coexistence of MLH3 germline variants in colon cancer patients belonging to families with Lynch syndrome-associated brain tumors. *Journal of Neuro-Oncology*.

[51] Duraturo F., Liccardo R., Cavallo A., Rosa M. D., Grosso M., Izzo P. (2011). Association of low-risk MSH3 and MSH2 variant alleles with Lynch syndrome: probability of synergistic effects. *International Journal of Cancer*.

[52] Huang J., Kuismanen S. A., Liu T. (2001). MSH6 and MSH3 are rarely involved in genetic predisposition to nonpolypotic colon cancer. *Cancer Research*.

[53] Lee S.-Y., Chung H., Devaraj B. (2010). Microsatellite alterations at selected tetranucleotide repeats are associated with morphologies of colorectal Neoplasias. *Gastroenterology*.

[54] Bandipalliam P. (2005). Syndrome of early onset colon cancers, hematologic malignancies & features of neurofibromatosis in HNPCC families with homozygous mismatch repair gene mutations. *Familial Cancer*.

[55] Herkert J. C., Niessen R. C., Olderode-Berends M. J. W. (2011). Paediatric intestinal cancer and polyposis due to bi-allelic PMS2 mutations: case series, review and follow-up guidelines. *European Journal of Cancer*.

[56] Poley J.-W., Wagner A., Hoogmans M. M. C. P. (2007). Biallelic germline mutations of mismatch-repair genes: a possible cause for multiple pediatric malignancies. *Cancer*.

[57] Plon S. E., Eccles D. M., Easton D. (2008). Sequence variant classification and reporting: recommendations for improving the interpretation of cancer susceptibility genetic test results. *Human Mutation*.

[58] Peters A., Born H., Ettinger R., Levonian P., Jedele K. B. (2009). Compound heterozygosity for MSH6 mutations in a pediatric lymphoma patient. *Journal of Pediatric Hematology/Oncology*.

[59] Kang S. Y., Park C. K., Chang D. K. (2015). Lynch-like syndrome: characterization and comparison with EPCAM deletion carriers. *International Journal of Cancer*.

[60] Buchanan D. D., Rosty C., Clendenning M., Spurdle A. B., Win A. K. (2014). Clinical problems of colorectal cancer and endometrial cancer cases with unknown cause of tumor mismatch repair deficiency (suspected Lynch syndrome). *Application of Clinical Genetics*.

[61] Boland C. R. (2013). The mystery of mismatch repair deficiency: lynch or lynch-like?. *Gastroenterology*.

[62] Mensenkamp A. R., Vogelaar I. P., Van Zelst-Stams W. A. G. (2014). Somatic mutations in *MLH1* and *MSH2* are a frequent cause of mismatch-repair deficiency in lynch syndrome-like tumors. *Gastroenterology*.

[63] Li F., Mao G., Tong D. (2013). The histone mark H3K36me3 regulates human DNA mismatch repair through its interaction with MutS*α*. *Cell*.

[64] Duraturo F., Liccardo R., Cavallo A., De Rosa M., Izzo P. (2013). *Synergistic Effects of Low-Risk Variant Alleles in Cancer Predisposition*.

[65] Iwahashi Y., Ito E., Yanagisawa Y. (1998). Promoter analysis of the human mismatch repair gene hMSH2. *Gene*.

[66] Ito E., Yanagisawa Y., Iwahashi Y. (1999). A core promoter and a frequent single-nucleotide polymorphism of the mismatch repair gene hMLH1. *Biochemical and Biophysical Research Communications*.

[67] Mrkonjic M., Raptis S., Green R. C. (2007). MSH2 −118T>C and MSH6 −159C>T promoter polymorphisms and the risk of colorectal cancer. *Carcinogenesis*.

[68] Raptis S., Mrkonjic M., Green R. C. (2007). MLH1 -93G>A promoter polymorphism and the risk of microsatellite-unstable colorectal cancer. *Journal of the National Cancer Institute*.

[69] Mao G., Lee S., Ortega J., Gu L., Li G.-M. (2012). Modulation of microRNA processing by mismatch repair protein MutL*α*. *Cell Research*.

[70] Zhang G., Gibbs E., Kelman Z., O'Donnell M., Hurwitz J. (1999). Studies on the interactions between human replication factor C and human proliferating cell nuclear antigen. *Proceedings of the National Academy of Sciences of the United States of America*.

[71] Shcherbakova P. V., Hall M. C., Lewis M. S. (2001). Inactivation of DNA mismatch repair by increased expression of yeast MLH1. *Molecular and Cellular Biology*.

[72] Marra G., Iaccarino I., Lettieri T., Roscilli G., Delmastro P., Jiricny J. (1998). Mismatch repair deficiency associated with overexpression of the MSH3 gene. *Proceedings of the National Academy of Sciences of the United States of America*.

[73] Landau D.-A., Slack F. J. (2011). MicroRNAs in mutagenesis, genomic instability, and DNA repair. *Seminars in Oncology*.

[74] Dong Y., Yu J., Ng S. S. M. (2014). MicroRNA dysregulation as a prognostic biomarker in colorectal cancer. *Cancer Management and Research*.

[75] Wang Y., Taniguchi T. (2013). MicroRNAs and DNA damage response: implications for cancer therapy. *Cell Cycle*.

[76] Valeri N., Gasparini P., Braconi C. (2010). MicroRNA-21 induces resistance to 5-fluorouracil by down-regulating human DNA MutS homolog 2 (hMSH2). *Proceedings of the National Academy of Sciences of the United States of America*.

[77] Svrcek M., El-Murr N., Wanherdrick K. (2013). Overexpression of microRNAs-155 and 21 targeting mismatch repair proteins in inflammatory bowel diseases. *Carcinogenesis*.

[78] Couch F. J., Rasmussen L. J., Hofstra R., Monteiro A. N. A., Greenblatt M. S., de Wind N. (2008). Assessment of functional effects of unclassified genetic variants. *Human Mutation*.

[79] Syngal S., Fox E. A., Li C. (1999). Interpretation of genetic test results for hereditary nonpolyposis colorectal cancer: implications for clinical predisposition testing. *JAMA*.

[80] Shaik A. P., Shaik A. S., Al-Sheikh Y. A. (2015). Colorectal cancer: a review of the genome-wide association studies in the kingdom of Saudi Arabia. *Saudi Journal of Gastroenterology*.

[81] Le Marchand L. (2009). Genome-wide association studies and colorectal cancer. *Surgical Oncology Clinics of North America*.

[82] Goldgar D. E., Easton D. F., Byrnes G. B., Spurdle A. B., Iversen E. S., Greenblatt M. S. (2008). Genetic evidence and integration of various data sources for classifying uncertain variants into a single model. *Human Mutation*.

[83] Smith T. R., Liu-Mares W., Van Emburgh B. O. (2011). Genetic polymorphisms of multiple DNA repair pathways impact age at diagnosis and TP53 mutations in breast cancer. *Carcinogenesis*.

[84] Lindor N. M., Rabe K., Petersen G. M. (2005). Lower cancer incidence in Amsterdam-I criteria families without mismatch repair deficiency: familial colorectal cancer type X. *JAMA*.

[85] Duraturo F., Liccardo R., Cavallo A., De Rosa M., Rossi G. B., Izzo P. (2015). Multivariate analysis as a method for evaluating the pathogenicity of novel genetic MLH1 variants in patients with colorectal cancer and microsatellite instability. *International Journal of Molecular Medicine*.

[86] Duraturo F., Cavallo A., Liccardo R. (2013). Contribution of large genomic rearrangements in Italian Lynch syndrome patients: characterization of a novel alu-mediated deletion. *BioMed Research International*.

[87] Muranen T. A., Mavaddat N., Khan S. (2016). Polygenic risk score is associated with increased disease risk in 52 Finnish breast cancer families. *Breast Cancer Research and Treatment*.

[88] Yuan J., Li Y., Tian T. (2016). Risk prediction for early-onset gastric carcinoma: a case-control study of polygenic gastric cancer in Han Chinese with hereditary background. *Oncotarget*.

[89] Talseth-Palmer B. A., Bauer D. C., Sjursen W. (2016). Targeted next-generation sequencing of 22 mismatch repair genes identifies Lynch syndrome families. *Cancer Medicine*.

